# Effects of lidocaine, dexmedetomidine, and their combination infusion on postoperative nausea and vomiting following laparoscopic hysterectomy: a randomized controlled trial

**DOI:** 10.1186/s12871-021-01420-8

**Published:** 2021-08-04

**Authors:** Siqi Xu, Shengbin Wang, Shenghong Hu, Xia Ju, Qing Li, Yuanhai Li

**Affiliations:** 1 Department of Anesthesiology, The Affiliated Anqing Hospital of Anhui Medical University, Anqing, 246000 China; 2 Department of Gynaecology and Obstetrics, The Affiliated Anqing Hospital of Anhui Medical University, Anqing, 246000 China; 3 Department of Anesthesiology, The First Affiliated Hospital of Anhui Medical University, Hefei, 230032 China

**Keywords:** Lidocaine, Dexmedetomidine, Postoperative nausea and vomiting, Laparoscopic hysterectomy

## Abstract

**Background:**

A few studies have reported that administration of lidocaine and dexmedetomidine relieves the incidence of postoperative nausea and vomiting (PONV). We explored whether combined infusion of lidocaine plus dexmedetomidine had lower occurrence of PONV undergoing laparoscopic hysterectomy with general anesthesia.

**Methods:**

A total of 248 women undergoing elective laparoscopic hysterectomy were allocated into the following four groups: the control group (group C, *n* = 62) received an equal volume of saline, the lidocaine group (group L, *n* = 62) received intravenous lidocaine (bolus infusion of 1.5 mg/kg over 10 min, 1.5 mg/kg/h continuous infusion), the dexmedetomidine group (group D, *n* = 62) received dexmedetomidine administration (bolus infusion of 0.5 µg/kg over 10 min, 0.4 µg/kg/h continuous infusion), and the lidocaine plus dexmedetomidine group (group LD, *n* = 62) received combination of lidocaine (bolus infusion of 1.5 mg/kg over 10 min, 1.5 mg/kg/h continuous infusion) and dexmedetomidine administration (bolus infusion of 0.5 µg/kg over 10 min, 0.4 µg/kg/h continuous infusion). The primary outcome was the incidence of nausea, vomiting, and PONV during the first 48 h after surgery. The secondary outcomes included the incidence of total 24 h PONV after surgery, intraoperative remifentanil requirement, postoperative pain visual analogue scale (VAS) scores and fentanyl consumption, the incidence of bradycardia, agitation, shivering, and mouth dry during post-anesthesia care unit (PACU) stay period.

**Results:**

The occurrence of nausea and PONV in group LD (5.0 and 8.3%) at 0–2 h after operation was lower than group C (21.7 and 28.3%) (*P* < 0.05). There was no statistically significant difference with respect to occurrence of nausea and PONV in groups L (13.3 and 20.0%) and D (8.3 and 13.3%) at 0–2 h after operation compared to group C (21.7 and 28.3%). The incidence of nausea, vomiting, and PONV at 2–24 and 24–48 h after surgery in all four groups was not statistically significant. The incidence of total 24 h PONV in group LD (33.3%) was significantly decreased compared to group C (60.0%) (*P* < 0.05). The cumulative consumption of fentanyl at 6 and 12 h after surgery was significantly reduced in group LD compared to other three groups (*P* < 0.05). The pain VAS scores were significantly decreased at 2, 6, and 12 h after operation in group LD compared to other three groups (*P* < 0.05). Remifentanil dose in the intraoperative period was significantly lower in groups LD and D compared with groups C and L (*P* < 0.05). The number of mouth dry, bradycardia, and over sedation during the PACU stay period was markedly increased in group LD (28.3, 30.0, and 35.0%, respectively) compared with groups C (1.7, 1.7, and 3.3%, respectively) and L (3.3, 5.0, and 6.7%, respectively) (*P* < 0.05).

**Conclusions:**

Lidocaine combined with dexmedetomidine infusion markedly decreased the occurrence of nausea and PONV at 0–2 h as well as the total 24 h PONV. However, it significantly increased the incidence of mouth dry, bradycardia, and over sedation during the PACU stay period after laparoscopic hysterectomy with general anesthesia.

**Trial registration:**

ClinicalTrials.gov (NCT03809923), registered on January 18, 2019.

## Background

Nausea and vomiting after surgery is one of unpleasant, trouble, and the most common side effects with general anesthesia. Laparoscopic surgery is widely used for gynecological patients because of some advantages, including postoperative pain relief and accelerated recovery after surgery [1]. It was reported that the incidence of PONV in high-risk patients with no prophylaxis was likely to reach up to 70 to 80% [2–4]. Nausea and vomiting after surgery may result in more discomfort and dissatisfaction for patients, disorder of electrolyte, and prolonged the time of hospitalization. Given the higher baseline risk (females, laparoscopic surgery, use of postoperative opioids, etc.), a single prophylactic measure is often not sufficient to achieve a satisfactory PONV prophylaxis.

Dexmedetomidine can generate sedation, analgesia, and minimal respiratory depression. Dexmedetomidine administration has several potential benefit effects, such as reducing catecholamine release [5], opioid-sparing [6], and improving the quality of recovery [7, 8]. It was revealed that systemic administration of dexmedetomidine might enhance the analgesic effect of opioids and reduce opioids requirement during the perioperative and postoperative period [9, 10], which might result in decreasing of opioid-related adverse reactions including PONV. Some studies have pointed that perioperative dexmedetomidine administration can reduce the incidence of PONV [11, 12]. In addition, some studies have showed that intravenous (IV) lidocaine has several beneficial effects including analgesic, anti-hyperalgesic, and anti-inflammatory properties [13, 14]. Recent meta-analysis showed that the perioperative lidocaine administration reduced risk of nausea but not vomiting during the first 48 h after operation [15]. Therefore, we hypothesized that the combination of lidocaine and dexmedetomidine would further reduce the incidence of nausea, vomiting, and PONV after laparoscopic hysterectomy. The primary purpose of the present study was to explore the effect of a combined application of dexmedetomidine plus lidocaine on nausea, vomiting, and PONV during the first 48 h after laparoscopic hysterectomy undergoing general anesthesia.

## Materials and methods

The present study was ratified by Ethics Committee of the Anqing Municipal Hospital (approval number: AQ042) and prospectively registered at www.clinicaltrials.gov (NCT03809923, date of registration: 18/01/2019). All methods were performed in accordance with the relevant guidelines and regulations in our present study. Patients were requested to written the informed consent before surgery. A total of 248 subjects who underwent laparoscopic total hysterectomy with general anesthesia were recruited. The inclusion criteria included American Society of Anesthesiologists (ASA) physical status I and II, 40–60 years of age, not taking the antiemetic drug which has an effect on the incidence of PONV within 24 h before surgery, and scheduled for elective laparoscopic hysterectomy. The exclusion criteria in the current study included obesity with BMI (body mass index) > 30 kg.m^−2^, preoperative atrioventricular block and bradycardia, history of allergy to local anesthetics, history of preoperative opioids medication and psychiatric, severe respiratory disease, and impaired kidney or liver function. The patients suffered urticaria, severe hypotension (mean arterial pressure [MAP] < 60 mmHg) or bradycardia (heart rate [HR] < 40 bpm), or arrhythmia during lidocaine and dexmedetomidine infusion period were excluded.

### Randomization and assigned groups

Randomization was performed using computer-generated random numbers, and patient allocation ratio was 1:1. Assignments were concealed in sequentially numbered opaque envelopes containing the group allocation 1 h before induction of anesthesia. Patients were allocated into four groups, including groups L, D, LD and C: patients in group L received bolus infusion of lidocaine (2%) 1.5 mg/kg over 10 min before induction of anesthesia, then lidocaine was infused at the rate of 1.5 mg/kg/h, and which was stopped 30 min before the end of operation [16]; patients in group D received bolus infusion of dexmedetomidine 0.5 µg/kg over 10 min before induction of anesthesia, then dexmedetomidine was infused at the rate of 0.4 µg/kg/h, and which was stopped 30 min before the end of operation [17]; patients in group LD received bolus infusion of lidocaine (2%) 1.5 mg/kg and dexmedetomidine 0.5 µg/kg over 10 min before induction of anesthesia, respectively, then lidocaine and dexmedetomidine were infused at the rate of 1.5 mg/kg/h and 0.4 µg/kg/h, and which was stopped 30 min before the end of operation; patients in group C received the same volume normal saline (0.9%) over 10 min before induction of anesthesia, then normal saline (0.9%) was continuous infused in equal volume, and which was stopped 30 min before the end of operation. All participants, including researchers, clinicians, nurses, and patients were all fully blinded to treatment allocation. The drug solutions were prepared by an anesthesiologist who was not blinded, but not otherwise involved in the study.

### Anesthesia protocol

All patients did not received any drugs before surgery. Patients routinely monitored mean blood pressure (MBP), heart rate (HR), electrocardiogram (ECG), end-tidal CO_2_ (PetCO_2_), and peripheral pulse oximeter (SPO_2_) after arriving at the operation room. Peripheral venous access of each patient was established by a nurse of operating room. To obtain sufficient oxygenation, 100% oxygen was given to each patient via facemask for 3 to 5 min before induction of anesthesia. Patients in the four groups were induced with target-controlled infusion (TCI) of propofol and remifentanil. The target predicted plasma concentration of propofol was set at 3.0 μg/mL [18], which was maintained for 3 min, followed by remifentanil TCI begun. The target predicted plasma concentration of remifentanil was set at 5.0 ng/ml [19]. Cis-atracurium 0.15 mg/kg was injected intravenously when the patients lost consciousness, and an endotracheal tube (ETT) with an internal diameter of 6.5 mm (female) was inserted into the trachea after adequate muscle relaxation. Mechanical ventilation was implemented using Fabius Draeger machine. Respiratory parameters were set as follows: tidal volume and respiratory rate were set 6–8 mL/kg and 12–14 beat/min (bpm) to maintain the PetCO_2_ between 35 and 45 mmHg during the intraoperative period, respectively. A supplemental dose of cis-atracurium was administered intermittently according to train of four (TOF) to maintain muscle relaxation during the anesthesia period.

The depth of anesthesia was monitored by bispectral index (BIS), and values were kept between 45 and 60 by adjusting plasma concentration of propofol during the anesthesia period, and hemodynamic variables within 20% of preoperative values. 30 min before the end of surgery, Fentanyl 1 μg/kg was administered intravenously, and then patients were connected to an IV patient-controlled analgesic machine (IVPCA) with 0.3 µg/kg/h fentanyl and 0.9% normal saline (100 ml of total volume) to deliver a bolus of 0.075 µg/kg of the above analgesics, with background continuous infusion of 2 mL/h, and a lockout time of 15 min. At the end of surgery, ondansetron 0.1 mg/kg was injected intravenously. Atropine (0.5 mg) and neostigmine (1 mg) was given when the patients restored spontaneous respiration. Endotracheal intubation was removed with TOF ratio at least 0.9. Thereafter, all patients were transported to the PACU. All patients were continued to observe for 2 h during the PACU stay period. The incidence of bradycardia, dry mouth, agitation, and shivering were recorded during the PACU stay period. The operations were performed by two high-experienced surgeons under a CO_2_ pneumoperitoneum, and the pressure of pneumoperitoneum was maintained between 10 and 12 mmHg for all patients. At the end of surgery, wound infiltration of surgical site with 10 ml ropivacaine (0.75%) was completed by one surgeon to provide additional the analgesia protocol after surgery for each patient.

### Outcomes variables

Our primary outcome was the incidence of nausea, vomiting, and PONV within the first 48 h after surgery. Nausea intensity was evaluated using a 10-point VAS (0 = no nausea, 10 = intolerable nausea) in the PACU and surgical ward. In the surgical ward, all patients were asked by an independent anesthesiologist who did not involve in the study to assess the incidence and extent of nausea according to the nausea VAS. We recorded the number of vomiting by direct asking the patients. Emetic episodes included vomiting and retching in the present study. The definition of vomiting after surgery was at least incident of vomiting or retching and PONV score was at least 4. The PONV was defined as patients who underwent events of nausea, vomiting or retching or combination of these. If patients underwent the following conditions such as sustaining nausea (more than 30 min) or vomiting or retching (great than or equal to 2 times), rescue antiemetics (ondansetron 8 mg or droperidol 1 mg) were given intravenously.

The secondary outcomes included the incidence of total 24 h PONV after surgery, the occurrence of bradycardia, dry mouth, agitation, and shivering during the PACU stay period, postoperative pain VAS scores, postoperative fentanyl consumption, as well as propofol and remifentanil dose during the anesthesia period, which were not registered outcomes in the clinicaltrials.gov. The intensity of pain after operation was estimated with a 10-cm VAS in the PACU and the ward (0 for no pain, 10 for the most imaginable pain). If postoperative VAS > 3, an additional 25 µg of fentanyl was treated intravenously until the VAS ≤ 3. Sedation levels of subjects during the PACU stay period were evaluated with the Ramsay sedation scale (1 = agitated and uncomfortable, 2 = co-operative and orientated, 3 = can follow simple directions, 4 = asleep but strong response to stimulation, 5 = asleep and slow response to stimulation and 6 = asleep and no response to stimulation). Sedation score ≥ 4 was regarded as excessive sedation. Bradycardia was defined as heart rate < 50 beats/min or a decrease more than 20% of baseline.

### Sample size calculation

Data from our preliminary study indicated that the occurrence of nausea, vomiting, and PONV within the first 24 h after surgery in the groups C, L, D and LD was 52, 38, 30, and 22%; 47, 35, 26, and 20%; 55, 45, 36, and 27%, respectively. We achieved Power analysis by PASS 11.0 with a β value set at 0.2 and α value set at 0.05. A sample size of 180, 203, and 216 was respectively needed, 54 subjects was allocated to each group, and considering rate of dropout, therefore, 62 patients were enrolled in each group for the present study.

### Statistical analysis

We used SPSS v.20 (IBM Corp., Armonk, NY, USA) software for completing statistical analyses in the present study. Data were expressed as the number or mean ± standard deviation. χ^2^ or Fisher’s exact test as appropriate was used for categorical data analysis. One-way analysis of variance (ANOVA) was used for continuous data analysis in all four groups. Repeated-measures ANOVA compared difference of the pain VAS scores and fentanyl consumption in the four groups during the first 24 h after surgery. If group differences were found by ANOVA to be significant, and Tukey’s post-hoc test was performed for further analyzed. If heterogeneity of variance was found, Dunnett’s T_3_ test was performed for further analyzed. There was statistical significant when *P* value < 0.05 apart from the post-hoc pairwise comparisons in which *P*-values were adjusted by Bonferroni correction.

## Results

**Fig. 1:**
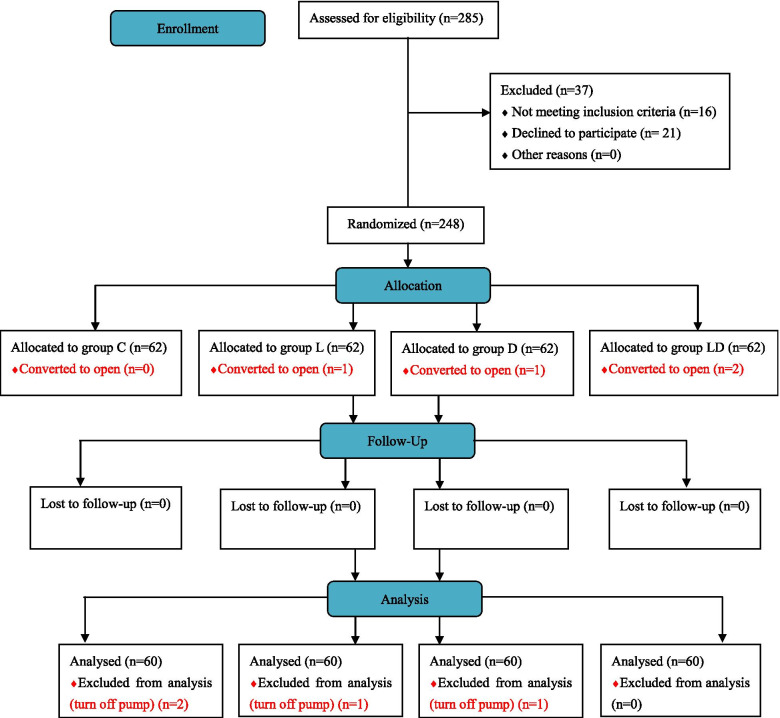
CONSORT flow diagram for the study

Of 285 patients screened for eligibility, 37 subjects were excluded because of preoperative bradycardia and refusing to participate the research. 248 subjects were randomized in the current study, four patients were converted to open surgery; four patients turned off the analgesic pump because of postoperative drastic vomiting. Eventually, 60 patients in each group were analyzed (Fig. 1).

There were no significant differences between the four groups regarding age, BMI, blood loss, intraoperative fluids, anesthesia time, operation time, history of smoking, history of PONV, history of motion sickness, and Apfel score for PONV risk (Table 1).

**Table 1:** Clinical data and intraoperative variables of patients

	**Group C (*n* = 60)**	**Group L (*n* = 60)**	**Group D (*n* = 60)**	**Group LD (*n* = 60)**	***P*-value**
Age (yr)	47.2 ± 5.1	45.4 ± 3.8	46.1 ± 4.3	46.6 ± 4.4	0.629
BMI	23.0 ± 1.4	22.8 ± 1.4	22.7 ± 1.5	22.5 ± 1.3	0.747
Operation time (min)	110.9 ± 9.0	113.4 ± 9.8	109.9 ± 11.1	114.3 ± 11.7	0.079
Anesthesia time (min)	133.6 ± 11.5	134.2 ± 10.9	132.8 ± 11.1	135.4 ± 11.7	0.707
Blood loss (ml)	74.4 ± 18.5	72.3 ± 16.9	73.4 ± 16.4	71.9 ± 14.3	0.187
Intraoperative fluids (ml)	893.8 ± 155.0	855.4 ± 135.1	904.3 ± 155.9	900.0 ± 158.4	0.379
ASA I/II, n	42/18	32/28	36/24	38/22	0.300
History of PONV, n (%)	6 (10.0%)	5 (8.3%)	7 (11.7%)	5 (8.3%)	0.912
History of motion sickness, n (%)	16 (26.7%)	18 (30%)	13 (21.7%)	20 (33.3%)	0.529
Smoking, n (%)	4 (6.7%)	3 (5.0%)	2 (3.3%)	3 (5.0%)	0.873
Intraoperative awareness, n (%)	0 (0%)	0 (0%)	0 (0%)	0 (0%)	
Apfel score for PONV risk, n (%)					
1	0 (0%)	0 (0%)	0 (0%)	0 (0%)	
2	3 (5.0%)	2 (3.3%)	1 (1.7%)	3 (5.0%)	0.736
3	36 (60.0%)	39 (65.0%)	34 (56.7%)	41 (68.3%)	0.560
4	21 (35.0%)	19 (31.7%)	25 (41.7%)	16 (26.7%)	0.364
Use of propofol (mg**·**kg^−1^**·**h^−1^)	5.5 ± 0.4	5.4 ± 0.3	4.9 ± 0.3^*✩^	4.4 ± 0.2^*✩#^	< 0.001
Use of remifentanil (μg**·**kg^−1^**·**h^−1^)	9.8 ± 0.6	9.4 ± 0.5^*^	5.7 ± 0.8^*✩^	5.3 ± 0.6^*✩#^	< 0.001

Data are present as mean ± standard deviation or number (%)

*Group C* Control group, *Group L* Lidocaine group, *Group D* Dexmedetomidine group, *Group LD* Lidocaine combined with dexmedetomidine group, *BMI* Body mass index, *ASA* American society of anesthesiologists, *PONV* Postoperative nausea and vomiting

^*^P versus Group C, ^✩^*P* versus Group L, ^#^*P* versus Group D

### The intraoperative requirement of propofol and remifentanil

The intraoperative requirement of propofol was lower in groups D and LD than groups C and L (*P* < 0.001, *P* < 0.001, *P* < 0.001, *P* < 0.001, respectively). The intraoperative requirement of remifentanil was lower in groups L, D, and LD than group C (*P* < 0.001, *P* < 0.001, *P* < 0.001, respectively). The intraoperative requirement of remifentanil significantly decreased in groups D and LD compared to group L (*P* < 0.001 and *P* < 0.001). The intraoperative requirement of propofol and remifentanil was the lowest in group LD (*P* < 0.05) (Table 1).

### The occurrence of nausea and vomiting after operation

The incidence of nausea and PONV during the first 0–2 h after operation was significantly lower in group LD than group C (3 (5.0%) and 13 (21.7%), *P* = 0.007; 5 (8.3%) and 17 (28.3%), *P* = 0.005, Bonferroni-corrected α = 0.0083 [0.05/6], respectively). In addition, the occurrence of total 24 h PONV was significantly decreased in group LD compared with group C (20 (33.3%) and 36 (60.0%), *P* = 0.003, Bonferroni-corrected α = 0.0083 [0.05/6]). There was not significant differences regarding incidence of nausea, vomiting and PONV during the 2–24 and 24–48 h after surgery, and the use of rescue antiemetics in the four groups. The prevalence of total 24 h PONV was not also significant differences in groups C, L, and D (36 (60.0%) and 28 (46.7%), *P* = 0.143; 36 (60.0%) and 24 (40.0%), *P* = 0.028; 28 (46.7%) and 24 (40.0%), *P* = 0.461, Bonferroni-corrected α = 0.0083 [0.05/6], respectively) (Table 2).

**Table 2:** Comparison of nausea and vomiting after operation

	**Group C (*n* = 60)**	**Group L (*n* = 60)**	**Group D (*n* = 60)**	**Group LD (*n* = 60)**	***P*-value**
Nausea n (%)					
0 to 2 h, n (%)	13 (21.7%)	8 (13.3%)	5 (8.3%)	3 (5.0%)^*^	0.031
2 to 24 h, n (%)	25 (41.7%)	23 (38.3%)	19 (31.7%)	17 (28.3%)	0.402
24 to 48 h n (%)	5 (8.3%)	4 (6.7%)	3 (5.0%)	2 (3.3%)	0.678
Vomiting n (%)					
0 to 2 h, n (%)	8 (13.3%)	7 (11.7%)	5 (8.3%)	3 (5.0%)	0.417
2 to 24 h, n (%)	18 (30.0%)	16 (26.7%)	15 (25.0%)	12 (20.0%)	0.648
24 to 48 h n (%)	2 (3.3%)	2 (3.3%)	1 (1.7%)	0 (0.0%)	0.523
PONV, n (%)					
0 to 2 h, n (%)	17 (28.3%)	12 (20.0%)	8 (13.3%)	5 (8.3%)^*^	0.025
2 to 24 h, n (%)	30 (50.0%)	26 (43.3%)	23 (38.3%)	19 (31.7%)	0.214
24 to 48 h n (%)	5 (8.3%)	5 (8.3%)	3 (5.0%)	2 (3.3%)	0.589
Total 24 h PONV, n (%)	36 (60.0%)	28 (46.7%)	24 (40.0%)	20 (33.3%)^*^	0.024
Rescue antiemetics, n (%)	12 (20.0%)	10 (16.7%)	8 (13.3%)	5 (8.3%)	0.311

Data are present as number (%)

*Group C* Control group, *Group L* Lidocaine group, *Group D* Dexmedetomidine group, *Group LD* Lidocaine combined with dexmedetomidine group, *PONV* Postoperative nausea and vomiting

^*^P versus Group C

### The pain VAS scores after surgery

The pain VAS scores were significantly lower at postoperative 2, 6 h in group L, and at postoperative 2, 6, and 12 h in groups D and LD compared to group C (*P* = 0.004, *P* = 0.030, *P* < 0.001, *P* < 0.001, *P* = 0.032, *P* < 0.001, *P* < 0.001, *P* < 0.001, respectively). We found that significant reduction of the pain VAS scores were observed at postoperative 2, 6, and 12 h in group LD compared to groups C, L, and D (*P* < 0.001, *P* < 0.001, *P* < 0.001, *P* < 0.001, *P* < 0.001, *P* < 0.001, *P* < 0.001, *P* < 0.001, *P* = 0.002, respectively). There was not statistical differences in all four groups regarding the pain VAS scores at postoperative 24 h (Table 3).

**Table 3:** The pain VAS scores after surgery

**VAS scores**	**Group C (*n* = 60)**	**Group L (*n* = 60)**	**Group D (*n* = 60)**	**Group LD (*n* = 60)**	***P*-value**	^**a**^***P***	^**b**^***P***	^**c**^***P***
Postoperative 2 h	3.1 ± 0.7	2.7 ± 0.7^*^	2.5 ± 0.7^*^	1.8 ± 0.6^*✩#^	< 0.001	0.004	< 0.001	< 0.001
Postoperative 6 h	3.0 ± 0.9	2.6 ± 0.8^*^	2.4 ± 0.8^*^	1.7 ± 0.6^*✩#^	< 0.001	0.030	< 0.001	< 0.001
Postoperative 12 h	2.5 ± 0.7	2.3 ± 0.8	2.1 ± 0.7^*^	1.6 ± 0.7^*✩#^	< 0.001	0.817	0.032	< 0.001
Postoperative 24 h	1.1 ± 0.7	1.0 ± 0.7	0.9 ± 0.6	0.9 ± 0.6	0.199	0.546	0.313	0.194

Data are present as mean ± standard deviation

*Group C* Control group, *Group L* Lidocaine group, *Group D* Dexmedetomidine group, *Group LD* Lidocaine combined with dexmedetomidine group, *VAS* Visual analogue scale

^*^P versus Group C, ^✩^*P* versus Group L, ^#^*P* versus Group D

^a^*P* for group C vs group L, ^b^*P* for group C vs group D, ^c^*P* for group C vs group LD

### The consumption of fentanyl

The consumption of fentanyl was significantly decreased in groups D and LD during the first 24 h after operation compared to group C (*P* = 0.001, *P* < 0.001, *P* = 0.001, *P* = 0.004, *P* = 0.001, *P* < 0.001, *P* < 0.001, *P* < 0.001, respectively). The consumption of fentanyl was lower at 6 h after surgery in group L than group C (*P* = 0.024). The consumption of fentanyl was much less at 6, and 12 h after surgery in group LD than group L (*P* = 0.003 and *P* = 0.033) (Table 4).

**Table 4:** Postoperative total fentanyl consumption during the first 24 h

**Total fentanyl consumption (**µg**)**	**Group C (*n* = 60)**	**Group L (*n* = 60)**	**Group D (*n* = 60)**	**Group LD (*n* = 60)**	***P*-value**	^**a**^***P***	^**b**^***P***	^**c**^***P***
Postoperative 2 h	45.5 ± 16.9	40.5 ± 12.4	36.5 ± 5.5^*^	36.3 ± 4.2^*^	< 0.001	0.331	0.001	0.001
Postoperative 6 h	125.4 ± 17.4	117.1 ± 13.4^*^	112.2 ± 11.7^*^	110.0 ± 7.1^*✩^	< 0.001	0.024	< 0.001	< 0.001
Postoperative 12 h	230.9 ± 22.0	222.0 ± 18.0	217.5 ± 15.0^*^	214.4 ± 10.2^*✩^	< 0.001	0.099	0.001	< 0.001
Postoperative 24 h	439.6 ± 29.1	429.9 ± 22.9	423.3 ± 20.4^*^	420.2 ± 16.5^*^	< 0.001	0.242	0.004	< 0.001

Data are present as mean ± standard deviation

*Group C* Control group, *Group L* Lidocaine group, *Group D* Dexmedetomidine group, *Group LD* Lidocaine combined with dexmedetomidine group

^*^P versus Group C, ^✩^*P* versus Group L

^a^*P* for group C vs group L, ^b^*P* for group C vs group D, ^c^*P* for group C vs group LD

### Adverse effects

The number of dry mouth, bradycardia, and excessive sedation was significantly higher during the PACU stay period in groups LD and D than group C (*P* < 0.001, *P* < 0.001, *P* < 0.001, *P* = 0.001, *P* < 0.001, *P* = 0.008, Bonferroni-corrected α = 0.0083 [0.05/6], respectively). Compared to group L, The number of dry mouth, bradycardia, and excessive sedation was significantly higher during the PACU stay period in group LD (*P* < 0.001, *P* < 0.001, *P* < 0.001, Bonferroni-corrected α = 0.0083 [0.05/6], respectively). The number of dry mouth was significantly higher during the PACU stay period in groups D than L (*P* = 0.001, Bonferroni-corrected α = 0.0083 [0.05/6]). Patients with agitation and shivering were not significant differences in the four groups (Table 5).

**Table 5:** Adverse events during the PACU period

**Adverse events**	**Group C (*n* = 60)**	**Group L (*n* = 60)**	**Group D (*n* = 60)**	**Group LD (*n* = 60)**	***P*-value**
Mouth dry, n (%)	1 (1.7%)	2 (3.3%)	15 (25%)^*✩^	17 (28.3%)^*✩^	< 0.001
Bradycardia, n (%)	1 (1.7%)	3 (5.0%)	12 (20.0%)^*^	18 (30.0%)^*✩^	< 0.001
Ramsay sedation score (≥ 4), n (%)	2 (3.3%)	4 (6.7%)	11 (18.3%)^*^	21 (35.0%)^*✩^	< 0.001
Patients with agitation, n (%)	11 (18.3%)	9 (15.0%)	5 (8.3%)	4 (6.7%)	0.162
Patients with shivering, n (%)	14 (23.3%)	12 (20.0%)	9 (15.0%)	7 (11.7%)	0.341

Data are present as number (%)

*Group C* Control group, *Group L* Lidocaine group, *Group D* Dexmedetomidine group, *Group LD* Lidocaine combined with dexmedetomidine group, *PACU* Post-anesthesia care unit

^*^P versus Group C, ^✩^*P* versus Group L

## Discussion

The main findings of the current research showed that combination of lidocaine and dexmedetomidine infusion only resulted in fewer incidence of nausea and PONV at 0–2 h after operation. However, it did not significantly reduce the incidence of nausea, vomiting, and PONV at 2–24 and 24–48 h after laparoscopic hysterectomy. In addition, we also found that lidocaine combined with dexmedetomidine administration significantly decreased the incidence of total 24 h PONV after operation.

Intraoperative lidocaine administration help to improve postoperative pain and reduces intraoperative opioid requirement. The lower overall opioid consumption may decrease the incidence of PONV. A study by Wang T et al. suggested that 1.5 mg/kg lidocaine bolus injection before induction of anesthesia, followed by continuous infusion at the speed of 2 mg/kg/h until the end of operation significantly reduced the occurrence of PONV at 6 h after gynecological laparoscopic surgery, but there was not comparable at 1 h and 24 h after operation. It might be associated with opioid-sparing and lower VAS scores [20]. Ahn E et al. indicated that lidocaine administration was significantly lower incidence of nausea compared to the control group with laparoscopic colectomy, it most likely attributed to intravenous lidocaine decreased the total amount of fentanyl [21]. The results of our study showed that intravenous lidocaine was lower postoperative pain VAS scores and fentanyl consumption within the first 6 h after surgery, but it did not reduce the incidence of nausea, vomiting, and PONV during the first 48 h after operation. Our findings were inconsistent with above studies. It might be due to the different dosages and time of lidocaine administration.

Intraoperative and postoperative use of opioids may induce the incidence of PONV. Minimum consumption of opioids may result in less opioid-related adverse events including PONV. Previous studies documented that dexmedetomidine decreased the occurrence of PONV by reducing requirement of opioids [22, 23]. Geng ZY et al. demonstrated that dexmedetomidine administration had lower incidence of nausea, which attributed to sympatholytic and opioid-sparing effect of dexmedetomidine [24]. The results from Li HJ et al. showed that dexmedetomidine reduced the incidence of nausea not vomiting at 2 h after operation [25]. In our study, we observed that dexmedetomidine administration significantly decreased remifentanil requirement, postoperative pain VAS scores, and consumption of fentanyl. However, dexmedetomidine infusion did not significantly decrease the incidence of nausea, vomiting, and PONV at 0–2, 2–24, and 24–48 h after operation compared with group C. It might be associated with the intensity of opioid-sparing effect of dexmedetomidine.

In a study by Bakan M et al. showed that intravenous lidocaine and dexmedetomidine infusion for laparoscopic cholecystectomy reduced the incidence of PONV undergoing patients requiring tracheal intubation for general anesthesia [26]. In our study, several identified independent risk factors for PONV such as female, history of smoking, motion sickness or PONV, and laparoscopic surgery in the current study were not comparable significance between the four groups. Our results indicated that combination of lidocaine and dexmedetomidine infusion significantly reduced nausea and PONV at 0–2 h as well as the incidence of total 24 h PONV after surgery. This might be associated with lidocaine plus dexmedetomidine administration resulted in less intraoperative remifentanil requirement and lower VAS pain scores during the first 24 h after surgery. We also found that combination of lidocaine and dexmedetomidine had no significant effect on nausea, vomiting, and PONV at 2–24 and 24–48 h. In addition, use of rescue antiemetics (ondansetron or droperidol) was not comparable in the four groups when patients underwent sustaining nausea (more than 30 min) or vomiting or retching (great than or equal to 2 times) after surgery.

In the present study, our results were explained by several probably reasons. First, combination of lidocaine and dexmedetomidine had better opioid-sparing effect than lidocaine and dexmedetomidine alone, which further reduced opioid consumption. Second, lidocaine plus dexmedetomidine infusion could provide better pain relief than lidocaine and dexmedetomidine alone after surgery, which resulted in less opioid consumption to achieve a lower incidence of opioid-related adverse events such as PONV. This was similar results by Xu et al. who found that lidocaine combined with dexmedetomidine infusion resulted in greater analgesic and opioid-sparing effect compared to lidocaine and dexmedetomidine infusion alone in patients undergoing abdominal hysterectomy [27]. Therefore, the combination regimen of lidocaine and dexmedetomidine had lower the rate of PONV, it might be due to better control postoperative pain and minimize opioid, which help to improve the quality of recovery and minimize opioid-related adverse events for facilitating enhanced recovery after surgery (ERAS).

Although dexmedetomidine possesses the hypnotic and sedative effect, bradycardia is the most common side effect of dexmedetomidine administration, especially a large dose of dexmedetomidine. Beloeil H et al. reported that dexmedetomidine-based, opioid-free anesthesia had higher rates of severe bradycardia [28]. A recent study reported prolonged recovery in patients receiving dexmedetomidine, which was explained by over-sedation, hypotension, and bradycardia [29]. Therefore, we chose a relatively lower dose (0.5 µg/kg loading, 0.4 µg/kg/h infusion) and shorten time of infusion for decreasing the incidence of bradycardia and facilitating recovery after the operation. Local anesthetic systemic toxicity (LAST) including neurotoxicity and cardiotoxicity remains a major concern for lidocaine infusion. Kaba A et al. showed that intravenous lidocaine (1.5 mg/kg loading, 1.5 mg/kg/h infusion) was lower than the toxic plasma concentrations of lidocaine [30]. In our study, a loading dose of lidocaine 1.5 mg/kg, given as an infusion over 10 min, follow by 1.5 mg/kg/h infusion, which was relatively safe. Lidocaine constant rate infusion (CRI) is associated with sedation [31]. Clinical trials proved that dexmedetomidine administration decreased the rate of agitation [32, 33] and shivering [34]. In our study, severe bradycardia was not observed during the combined infusion of lidocaine plus dexmedetomidine and dexmedetomidine alone. The results of our study demonstrated that the incidence of bradycardia, over sedation, and dry mouth were much higher in group LD than group C during the PACU stay period. In addition, although the incidence of agitation and shivering decreased in group LD, it was not significant difference between the two groups.

Our study has several limitations. On the one hand, our sample was relatively small, the incidence of the total 24 h PONV was higher than we expected in the group D compared to group C, therefore, we did not find a significant statistic difference in group D and group C. On the other hand, although we speculated that combined application of lidocaine plus dexmedetomidine further inhibited stress response, we were not detected levels of catecholamine. Finally, the same dose of ondansetron was treated when patients underwent sustaining nausea (more than 30 min) or vomiting or retching (great than or equal to 2 times) after surgery, which might have effect on the incidence of PONV.

## Conclusions

Lidocaine combined with dexmedetomidine administration might provide better reduction of early nausea, PONV and the total 24 h PONV in patients undergoing laparoscopic hysterectomy. It had no significant effect on nausea, vomiting, and PONV during the 2–24 and 24–48 h. This suggested that the antiemetic effects of lidocaine plus dexmedetomidine might be rather short-lived. However, it increased the incidence of bradycardia, dry mouth, and over sedation during the PACU stay period. Therefore, the combination regimen of lidocaine and dexmedetomidine had lower the rate of PONV, it might be due to better control postoperative pain and minimize opioid, which help to improve the quality of recovery and minimize opioid-related adverse events for facilitating enhanced recovery after surgery (ERAS).

## Data Availability

The datasets used and/or analysed during the current study are available from the corresponding author on reasonable request.

## Abbreviations

PONV: Postoperative nausea and vomiting
PACU: Post-anesthesia care unit
VAS: Visual analogue scale
ERAS: Enhanced recovery after surgery
IV: Intravenous
ASA: American society of anesthesiologists
MBP: Mean blood pressure
HR: Heart rate
ECG: Electrocardiogram
PetCO_2_: End-tidal CO_2_
SPO_2_: Peripheral pulse oximeter
TCI: Target-controlled infusion
ETT: Endotracheal tube
TOF: Train of four
BIS: Bispectral index
IVPCA: Intravenous patient-controlled analgesic machine
ANOVA: Analysis of variance
BMI: Body mass index
CRI: Constant rate infusion
TIVA: Total intravenous anesthesia
LAST: Local anesthetic systemic toxicity
